# Intensity-based optoretinography reveals sub-clinical deficits in cone function in retinitis pigmentosa

**DOI:** 10.3389/fopht.2024.1373549

**Published:** 2024-06-04

**Authors:** Mina Gaffney, Thomas B. Connor, Robert F. Cooper

**Affiliations:** ^1^ Joint Department of Biomedical Engineering, Marquette University and the Medical College of Wisconsin, Milwaukee, WI, United States; ^2^ Ophthalmology & Visual Sciences, Medical College of Wisconsin, Milwaukee, WI, United States

**Keywords:** retinitis pigmentosa, adaptive optics scanning light ophthalmoscopy, optoretinography, microperimetry, cone photoreceptors

## Abstract

**Introduction:**

Clinical tools have been widely used in the diagnosis, description, and monitoring the progression of retinitis pigmentosa (RP); however, many of these methods have inherently low sensitivity and specificity, and significant photoreceptor disruption can occur before RP progression has clinically manifest. Adaptive optics scanning light ophthalmoscopy (AOSLO) has shown promise as a powerful tool for assessing photoreceptor disruption both structurally and functionally due to its increased resolution.

**Methods:**

Here we assess photoreceptor structure and function at the cellular level through AOSLO by acquiring intensity based optoretinography (iORG) in 15 individuals with no reported retinal pathology and 7 individuals with a prior clinical diagnosis of RP. Photoreceptor structure was quantified by calculating cone nearest neighbor distance (NND) across different retinal eccentricities from the AOSLO images. Cone outer segment length was measured across different retinal eccentricities using optical coherence tomography (OCT) derived longitudinal reflectivity profiles (LRPs). Finally, iORG measures of photoreceptor function were compared to retinal sensitivity as measured using the macular integrity assessment (MAIA) microperimeter.

**Results:**

Broadly, participants with RP exhibited increasing cone nearest neighbor distances and decreasing cone outer segment length as a function of retinal eccentricity, consistent with prior reports for both controls and individuals with RP. Nearly all individuals with RP had reduced iORG amplitudes for all retinal eccentricities when compared to the control cohort, and the reduction was greater in eccentricities further from the fovea. Comparing iORG amplitudes to MAIA retinal sensitivity, we found that the iORG was more sensitive to early changes in photoreceptor function whereas MAIA was more sensitive to later stages of disease.

**Discussion:**

This highlights the utility of iORG as a method to detect sub-clinical deficits in cone function in all stages of disease progression and supports the future use of iORG for identifying cells that are candidates for cellular based therapies.

## 1 Introduction

Retinitis pigmentosa (RP) is a group of inherited retinal diseases which can lead to photoreceptor degeneration and concomitant progressive loss of peripheral vision which encroaches on the central retina over time (1, 2). RP is the most common inherited retinal disease, impacting approximately one in 4000 individuals globally (1, 3, 4). Functionally, RP leads to reduced vision in low light conditions, reduced peripheral vision, color vision deficiencies, and eventually reduced visual acuity and legal blindness. Mutations in more than 87 genes lead to RP, and there are three main inheritance patterns: autosomal dominant, autosomal recessive, and X-linked (2). Previous studies have reported that disease progression is mediated at least in part by the genetic subtype of RP (5).

Current techniques for diagnosis and monitoring of RP rely heavily on clinical imaging to assess retinal structure. Specifically, fundus imaging can reveal telltale signs of RP such as bone-spicule hyperpigmentation, narrowing of retinal arterioles, cystoid macular edema, and a yellowed appearance of the optic disk. Optical coherence tomography (OCT) has revealed even more structural disruptions in RP including inner retinal thickening, photoreceptor layer and retinal pigment epithelium thinning, and cystoid macular edema (6–8). While these imaging techniques are useful for diagnosing RP, they are generally used after the patient has already reported significant visual symptoms.

Clinical tools such as electroretinography, microperimetry, Humphrey visual field testing, and visual acuity testing have been used to characterize and monitor the functional impact of RP on the retina (9–18). By their nature, these functional assays evaluate large areas of the retina that span hundreds of photoreceptors. Like their structural counterparts, they lack the sensitivity required for monitoring early disruptions in retinal cells, meaning significant damage can occur before manifesting clinically. Indeed, previous studies investigating the relationship between foveal cone metrics such as density, spacing and visual acuity have consistently shown that visual acuity remained within a normal range until there was a 40% - 60% reduction in foveal cones (19, 20).

With the recent advancements in gene therapy, cell replacement, and small molecule strategies showing promising results for future use in the treatment of inherited retinal diseases (21–24) more sensitive tests of photoreceptor function are needed to better monitor disease progression and test treatment efficacy. Optoretinography (ORG) is a non-invasive method of assessing photoreceptor function *in vivo* using only the light backscattered from retinal cells (25–28). There are at present two methods of obtaining ORGs: phase-based optoretinography (pORGs), and intensity-based optoretinography (iORGs). pORGs are determined using the argument (or phase) of the complex OCT signal, and are typically calculated by determining the change in phase as referenced to the cone boundary. This is then related to the change in optical path length (29–35). Conversely, iORGs are created from changes in the amplitude of backscattered light from photoreceptors using adaptive optics flood illumination ophthalmoscopes (26, 36) adaptive optics scanning light ophthalmoscopes (AOSLOs) (25, 37, 38), and OCTs (39–43). The spatial and cellular selectivity of optoretinography makes it a particularly attractive technique for assessing photoreceptor disease. Indeed, prior work using pORGs in three individuals with autosomal recessive RP showed decreased function in photoreceptors (44). This predicates a broader question about the utility of the ORG for the clinic, and by extension its relationship to existing clinical tools.

In this work, intensity-based optoretinography is used to evaluate photoreceptor function in individuals with retinitis pigmentosa and controls with no reported retinal pathology. Additionally, this work examines the iORG’s relationship to clinical microperimetry and metrics of photoreceptor mosaic structure across multiple retinal locations. The relationship between each of these measurements is then compared to eccentricity-matched control data.

## 2 Materials and methods

### 2.1 Research participants

This study was approved by the Institutional Review Boards at the Medical College of Wisconsin (PRO00038673) and was conducted in accordance with the tenets of the Declaration of Helsinki. 15 control participants (n=4 assigned male at birth; n=11 assigned female at birth) with a mean (± standard deviation) age of 30.7 ± 10.6 years and 9 individuals with RP were recruited from clinical referrals and individuals that participated in prior studies. Of the 9, only 7 individuals (n=3 assigned male at birth; n=4 assigned female at birth) with a mean (± standard deviation) age of 52.86 ± 10.54 years were included in the final analysis of this study due to poor image quality in the 2 excluded individuals. Informed consent was obtained from all study participants after the possible risks of the study were explained. An ocular health questionnaire was used to determine study eligibility and obtain a self-reported ocular history from each participant. Each participant also underwent an eye exam with a physician at the Froedtert Eye Institute (T.C.) and their ocular biometry was measured using an IOLMaster (Carl Zeiss Meditec, Dublin CA). Exclusion criteria for control participants included self-reported ocular or systemic disease with the potential to impact ocular health (e.g. diabetes, hypertension). For individuals with RP, exclusion criteria included any additional self-reported ocular or systemic diseases which impact ocular health besides RP, as in control participants. The study eye of each participant was chosen at random after enrollment in the study. In some cases, the study eye was changed after the ocular health questionnaire, eye exam, or initial clinical imaging revealed prior injury or significant fixation deficit in the originally selected study eye (n=3).

### 2.2 Adaptive optics scanning light ophthalmoscopy (AOSLO) and stimulus delivery

Adaptive optics scanning light ophthalmoscopy (AOSLO) was used to characterize photoreceptor structure and function in each participant. Prior to AOSLO imaging, mydriasis and cycloplegia were accomplished by using one drop each of tropicamide (1%) and phenylephrine (2.5%) in the study eye of each participant.

A custom Apaeros AOSLO (Boston Micromachines Corporation, Cambridge MA, USA) was used to acquire videos of the photoreceptor mosaic using four simultaneous imaging channels. Three of the imaging channels (confocal, direct, and reflect) use a low coherence (1.8 µm) 850 ± 60 nm superluminescent diode (Superlum, Cork, Ireland) for the illumination source. The fourth imaging channel acquires confocal images using a high coherence (674 µm) 760 ± 0.05 nm laser diode (ThorLabs Inc., Newton NJ, USA). A 780 nm superluminescent diode (Superlum, Cork, Ireland) was used for wavefront sensing and a 97-actuator deformable mirror (ALPAO, Montbonnot, France) was used to correct the higher-order aberrations within the wavefront of each participant’s eye. A 554 ± 45 nm (17.6 µW/degree^2^) Maxwellian-view source focused to ~1mm at the cornea was used to deliver stimuli. The three low coherence imaging channels were used to capture structural images of the retina. The high coherence imaging channel was used to capture intensity-based optoretinography data.

Photon density at the retina was estimated for each subject using previously reported approach (29). In brief, the spectrum and power of the stimulus source at the cornea were measured using a spectrometer (Mavospec Base, Gossen Foto – und Lichtmesstechnik, Nürnberg Germany) and power meter (Thorlabs, Newton NJ). Planck’s constant (6.626e-34 J.s.) and the speed of light in a vacuum (3.0e8 m/s) were then used to convert the power of the stimulus source at the retina from watts to photons per second. From there, the absorption by the lens and macular pigment were estimated using previously reported values that correspond to each subject’s age at imaging (45, 46). The absorption values were then converted to transmission and multiplied by the stimulus photons per second. To account for the stimulus area, photons per second were divided by the stimulus area in degrees (1.13 degree^2^) resulting in photons per second per degree^2^. Multiplying by the stimulus duration (66ms), retinal magnification factor (291µm per degree) (47), and the ratio of the subject’s axial length to a reference 24mm axial length resulted in a final value in terms of photons per µm^2^.

#### 2.2.1 AOSLO structural imaging and montaging

To confirm the retinal locations of the targeted stimulus locations, we collected structural images of photoreceptors using the confocal and split detection (48) modalities of our AOSLO in a 2x2° box surrounding the fovea using a 1x1°field of view, and two strips going out to 8–10° temporal and superior to the fovea using a 1.5x1.5°field of view. After data acquisition, all structural videos were registered using the Apaeros software. Intra-frame distortion and torsion was mitigated in each video using a previously described approach (49) using custom software. Briefly: we corrected for residual distortions present in the video by finding the median translation required to align all frames of the image sequence to each strip of the reference frame. The inverse of the median translation was applied to all frames in the video. All aligned, distortion-corrected frames from each location were averaged to create a single image.

All registered images were montaged using a previously described automatic montaging script (49) (https://github.com/BrainardLab/AOAutomontaging), and the resultant montage was validated in Adobe Photoshop CC where each layer contained a single montaged AOSLO image. The two structural modalities of interest, confocal and split detection, were co-registered and grouped by modality.

We extracted bound nearest neighbor distance (NND) from the same coordinates used for iORGs using a previously described approach (50) and software (https://github.com/OCVL/Metricks).

#### 2.2.2 Acquiring and extracting intensity-based optoretinography (iORG) signals

##### 2.2.2.1 iORG data acquisition

To test the function of individual cone photoreceptors, the AOSLO described above was used to collect iORG data from three (1.5, 4, 8°) or four (1, 2, 4, 8°) retinal locations temporal from the participants’ preferred retinal locus (PRL). For individuals with RP, all retinal locations up to 8° temporal were attempted. However, individuals with intermediate to advanced RP often did not have intact retina at these eccentricities; in these individuals, we imaged all eccentricities possible, and the edge of the transition zone (TZ) as determined during the data acquisition for the structural montage. The edge of the lesion was defined as the area of the retina in which cone inner segment structures were no longer visible using our split detection modality (48). This pattern was chosen to sample what are ostensibly the “most healthy” photoreceptors (furthest from edge of the TZ) as well as the “least healthy” photoreceptors (closest to the edge of the TZ).

Videos were collected using a 29.4 Hz framerate using a 1x1°field of view. The following nomenclature, which was used in our previous iORG study (25), will be used to describe our experiment:

- **Acquisition:** A single AOSLO video.
- **Trial:** A series of 10 iORG acquisitions.
- **Permutation:** A group of trials taken across different retinal eccentricities. The order of the eccentricities was selected randomly.

**Figure 1:**
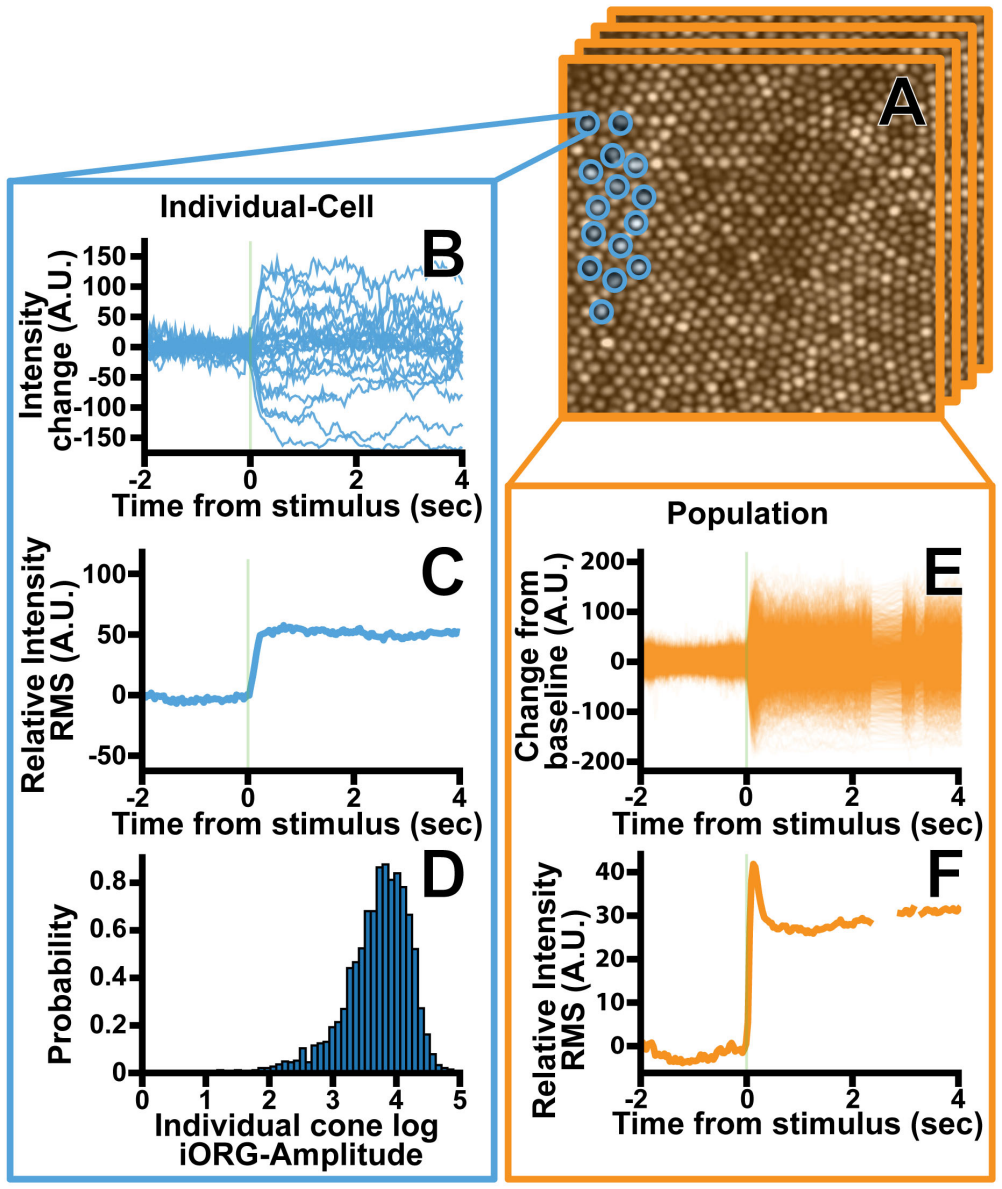
Overview of the iORG extraction process for an individual cone photoreceptor. **(A)** Individual photoreceptor centers were identified semi-automatically (blue) a column is then projected through each cone and the average pixel intensity within the column is calculated for each frame. **(B)** The average framewise intensity for a given cone is then plotted across time for all stimulus acquisitions in a given trial. **(C)** The iORG data is summarized by calculating the moving root mean square and subtracting the pre-stimulus mean from each signal. **(D)** The log iORG amplitude for all cones at a given imaging location were then plotted in a probability density function. **(E)** In order to assess how an entire population of cones behave in a given trial, the average intensity across all cones within a frame was plotted across time for all stimulus acquisitions in a given trial. **(F)** The population iORG data is summarized by calculating the moving root mean square and subtracting the pre-stimulus mean from each signal.

Each acquisition consisted of six seconds of recording with a 68ms stimulus delivered two seconds into the video. (**Figure 1A**) A three-minute dark adaptation period preceded all trials. After one control trial, three stimulus trials were conducted.

##### 2.2.2.2 Video post-processing

After all trials were collected, all individual acquisitions were registered using the Apaeros registration software. The reference frame used for registration was then chosen automatically from the low coherence structural imaging channel. Each frame within a given video was then aligned to the reference frame using normalized cross correlation (NCC) based strip-registration, and all other channels were aligned using the transform determined using the reference channel. A NCC “threshold” was used to exclude frames with poor strip alignment to the reference. As the goal of registration in this study was to align as many frames to the reference frame as possible, a low NCC threshold was used (0.1). All acquisitions were manually inspected and registered videos with low frame numbers, registration errors, or poor SNR were registered again by manually adjusting registration parameters until the successfully registered number of frames per video was maximized.

Once strip registration was complete, a fully automated pipeline script was used to further process the high coherence (iORG) imaging channel. This script first excludes registered acquisitions with less than half of their original frames. Intra-frame distortion caused by eye motion was mitigated using approach described above (49). Following distortion removal, full-frame drift caused by transverse chromatic aberration and residual torsion were removed by performing an optimization-based full-frame affine registration of each frame in the video to the original reference frame. This registration used correlation as a metric, regular step gradient-descent as an optimizer, and an affine transformation model.

Finally, all registered, pipelined acquisitions from each retinal location were averaged to create a super-average image for each location. A semi-automated cone detection algorithm was then used to identify the pixel coordinates of each cone center in each super-average image (*Mosaic*; Translational Imaging Innovations, Hickory NC). In individuals with RP, we used a co-located split-detection image to validate that our coordinates corresponded to cone centers.

##### 2.2.2.3 Extracting iORG signals

To generate an iORG waveform from each co-registered acquisition, we first standardized image-containing regions of each frame of each video to a mean of 0 and a standard deviation of 1. All frames were then rescaled to a mean of 70 and a standard deviation of 35; these values were empirically chosen based on the typical mean and average of our data. This step was completed to: minimize the effect of inter and intra-acquisition variability in image statistics due to image-wide changes in intensity, to keep all images in terms of image (not standardized) units, and to facilitate an equitable comparison between participants.

Coordinate positions for each cone were refined to each acquisition’s average image using a hill climbing algorithm to account for residual inter-video registration errors. Next, iORG signals were created by projecting a cylindrical column thorough each frame of a given acquisition and averaging the pixel intensity values for each frame. (**Figure 1A**) The size of the column was automatically determined based on the underlying nearest-neighbor cone spacing of the coordinates and encompassed the central full width half max of each cone’s reflectance profile. Individual cone signals were excluded from further analysis if more than 50% of the frames within 0.2 seconds of stimulus delivery were absent or 50% of the frames were missing from the acquisition as a whole, as missing data during that time would mean we could not guarantee that the cone had received the stimulus. Moreover, cones were excluded from analysis if they had less than 50% of the total number of acquisitions for a given location. Finally, the pre-stimulus mean of the remaining cone signals was subtracted from each signal. (**Figures 1C,E**).

##### 2.2.2.4 Summarizing multiple iORGs with RMS

The iORG signals were summarized by taking a frame-wise RMS of all cones in a single acquisition (38) (population-RMS; Equation 1; **Figures 1E, F**), or of a single cone across all of its acquisitions (25) (individual-RMS; Equation 2; **Figures 1B, C**). Population-RMS for each frame index is therefore defined as:


(1) $$RMS_{pop}\left[t\right]=\;\sqrt{\frac{1}{n}\sum_{1}^{n}R_{c}{\left[t\right]}^{2}}$$

where $R_{c}$ is the mean-subtracted reflectance of cone *c* at frame index *t* for the number of cones n , and individual-RMS is defined as:


(2) $$RMS_{indiv}\left[t\right]=\;\sqrt{\frac{1}{m}\sum_{1}^{m}R_{a}{\left[t\right]}^{2}}$$

Where *R_a_* is the mean-subtracted reflectance of a single cone’s acquisition *a* at frame index *t* for all *m* acquisitions. Equation 2 was repeated for each cone. For both iORG RMS types (i.e. population-RMS or individual-RMS) we extracted the amplitude, which we defined as the pre-stimulus mean RMS within one second before stimulus delivery subtracted from the 99^th^ percentile of the RMS within one second after stimulus delivery. We determined that the standard deviation of individual-RMS amplitudes was proportional to their mean, indicating a log transform (51). Thus, the amplitude of each individual-RMS was log transformed (**Figure 1D**), and all cones log individual-RMS amplitudes from each location were summarized with a cumulative distribution function.

### 2.3 Measuring outer segment length using optical coherence tomography (OCT)

We obtained nominally 6mm or 9mm HD Line scans from each participant in the study eye using a Cirrus (Carl Zeiss Meditec, Dublin CA) OCT. OCT Reflectivity Analytics (https://github.com/wilkb777/ORA), a previously described longitudinal reflectance profile (LRP) analysis tool (52) was then used to measure cone outer segment length at the same retinal eccentricities imaged for iORG by calculating the distance between the peaks in the longitudinal reflectance profile that correspond to the ellipsoid zone and the interdigitation zone.

### 2.4 Macular integrity assessment (MAIA) microperimetry

Microperimetry was used to assess retinal health in the study eye of each participant using Macular Integrity Assessment (MAIA; iCare, Vantaa, Finland) while un-dilated. The non-study eye of each participant was occluded. The room lights were off during the test; however, there was no dark adaptation prior to testing. Fixational stability and the PRL were measured by instructing the participant to fixate on the center fixation target within the device for 10 seconds. The stimulus delivery locations were centered about the PRL. A customized protocol was performed using a “4–2” testing strategy, a Goldmann III spot size, and locations centered at the PRL, 0.5°, 1°, 2°,3°, 4°, 5°, 6°, and 8° away from the PRL in each of the cardinal directions. Stimulus intensity values ranged from 0 to 36 dB, with 0 dB being the brightest stimulus and 36 dB being the dimmest.

After the exam was complete, final sensitivity results were overlaid onto the reference fundus image with the stimulus intensity threshold for each test location expressed in dB. The reference fundus image and sensitivity results were then exported for further analysis. Finally, all AOSLO montages were co-registered with the fundus image, and the retinal sensitivity closest to the iORG sample locations were used as the retinal sensitivity for that location.

## 3 Results

### 3.1 Demographics and imaging success

We obtained cone photoreceptor iORGs across 15 individuals with no reported retinal pathology, and successfully analyzed iORGs from all attempted locations (three locations: n=5, four locations: n=10) but one (13604: 4°). Of the nine individuals with RP recruited, two individuals were excluded from further analysis due to poor image quality due to cataracts or substantial retinal edema. In the remaining cohort, we successfully obtained and analyzed cone iORGs in all locations with an intact photoreceptor mosaic (median: 3 locations, range 1–4). Moreover, we successfully obtained iORGs either from the temporal edge of their remaining photoreceptor mosaic, or 8° temporal from their PRL. When possible, the clinical RP diagnosis as well as the genetic mechanism was confirmed using previously acquired genetics results (**Table 1**).

**Table 1:** Participant demographics and stimulus photon density delivered to the retina.

Subject ID	Study eye	Diagnosis	Inheritance pattern	Sex at birth	Age (Years)	Axial length (mm)	Photon density (photons/µm^2^)
4710	OS	None	none	F	30	24.11	2.37E+07
8941	OD	None	none	F	21	23.5	2.53E+07
10397	OD	None	none	F	25	25.74	2.09E+07
13604	OD	Amblyopia OS	none	F	30	22.28	2.77E+07
15077	OD	None	none	F	26	23.92	2.42E+07
17575	OS	None	none	F	40	25.27	2.11E+07
23045	OD	None	none	F	26	22.99	2.62E+07
23521	OD	None	none	M	64	25.99	1.86E+07
33388	OS	None	none	M	36	23.15	2.54E+07
36828	OD	None	none	M	35	24.46	2.28E+07
50093	OS	None	none	M	24	25.21	2.19E+07
54784	OD	None	none	F	24	23.06	2.61E+07
56450	OS	None	none	F	31	24.7	2.25E+07
64774	OS	None	none	F	25	23.77	2.45E+07
88735	OS	None	none	F	24	23.67	2.48E+07
13090	OS	Retinitis Pigmentosa	USH2A, recessive	F	67	24.2	2.12E+07
15660	OD	Retinitis Pigmentosa	Not Reported	M	48	23.57	2.38E+07
33948	OD	Retinitis Pigmentosa	Unknown mutation	M	36	23.44	2.48E+07
53885	OD	Retinitis Pigmentosa	RP, dominant RHO mutation	F	61	25.11	2.02E+07
60151	OD	Ushers Syndrome	USH2A, recessive	F	45	23.14	2.49E+07
64010	OD	Retinitis Pigmentosa	RP1, dominant	F	55	23.29	2.39E+07
89385	OS	Retinitis Pigmentosa	Not Reported	M	58	25.07	2.04E+07

### 3.2 Cone structure and retinal eccentricity

**Figure 2:**
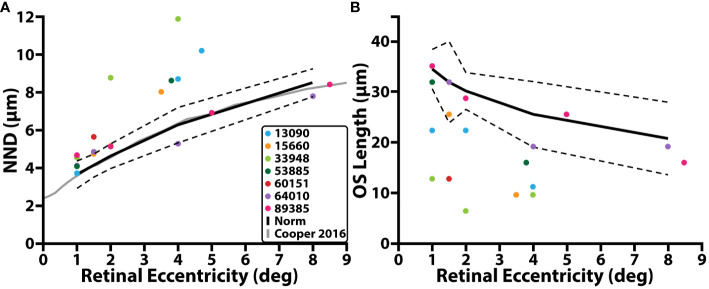
Cone structure and retinal eccentricity. **(A)** NND (µm) as a function of retinal eccentricity temporal with respect to the fovea. **(B)** Cone outer segment (OS) length as a function of retinal eccentricity. Individuals with RP are denoted by colored circles, average control data is denoted by the solid black line, dashed black line represents 95% prediction interval, solid grey line represents average control data from a previous study.

Cone appearance, NND, and outer segment lengths in our control population were consistent with previously published normative data. The 95% confidence interval of our NND control population (**Figure 2**; black dashed line) fully overlapped with our previously published average data (**Figure 2**; grey solid line) (50). The cone outer segment lengths measured in this study for the control cohort (range: 32µm – 38.4µm at 1°, range: 25.6µm – 35.2µm at 1.5°, range: 27.2µm -32µm at 2°; range: 19.2µm – 32µm at 4°, and range: 12.8µm – 28.8µm at 8°) fell within the range of previously published reports (52–55).

In individuals with RP, confocal AOSLO images from the peri- and para-fovea (>2° eccentricity) showed both abnormally waveguiding cones and a mottled mosaic appearance. Split-detection images of the same cells showed intact inner segments (**Supplementary Figure 1**). NND at these locations was greater than that of the control cohort while OS length is shorter, and nearly all locations (5/7; mean NND z-score = 3.93, range = 0.08 – 11.68; mean OS length z-score = 3.44, range = 0.35 – 6.57) were outside their respective normative 95% prediction intervals (**Figure 2**, black dashed line; **Table 2**). Near the fovea (<=2° eccentricity), confocal AOSLO images featured far more normally waveguiding cones (Gaussian profiles; **Supplementary Figure 1**). OS length at these locations consistently fell outside our normative prediction interval (8/11; mean z-score = 6.23, range = 0.516 – 10.64), but NND did not (5/11; mean z-score = 2.80, range = 0.04 – 12.10).

**Table 2:** Z-scores for nearest neighbor distance (NND), cone outer segment (OS) length, and population-RMS amplitude for all individuals with RP and the control means and standard deviations used to calculate the Z-scores.

Subject ID	Metric	1°	1.5°	2°	4°	8°
**13090**	**NND**	0.23		1.63	5.09	
	**OS length**	10.64		6.94	6.57	
	**iORG**	1.74		7.29	4.10	
**15660**	**NND**		1.84		3.69	
	**OS length**		1.5		3.7	
	**iORG**		2.97		3.63	
**33948**	**NND**	2.36		12.1	11.68	
	**OS length**	10.64		8.30	3.7	
	**iORG**	2.88		8.56	4.00	
**60151**	**NND**		4.44			
	**OS length**		9.5			
	**iORG**		3.32			
**64010**	**NND**		0.043		2.88	1.66
	**OS length**		2.5		0.83	0.63
	**iORG**		1.00		2.58	3.24
**53885**	**NND**	1.16			4.93	
	**OS length**	8.69			6.57	
	**iORG**	2.07			4.08	
**89385**	**NND**	2.70		1.55	1.39	0.08
	**OS length**	3.03		0.52	0.35	2.02
	**iORG**	1.49		2.58	0.55	1.63
**Control Mean**	**NND**	3.64	4.13	4.62	6.26	8.47
	**OS length**	30.2	28.0	26.0	22.0	16.9
	**iORG**	36.8	33.0	33.9	34.7	28.04
**Control Standard Deviation**	**NND**	0.39	0.35	0.35	0.48	0.39
	**OS length**	1.6	1.6	2.36	3.34	3.63
	**iORG**	8.71	7.53	3.03	7.35	6.75

### 3.3 iORG RMS across retinal eccentricity

**Figure 3:**
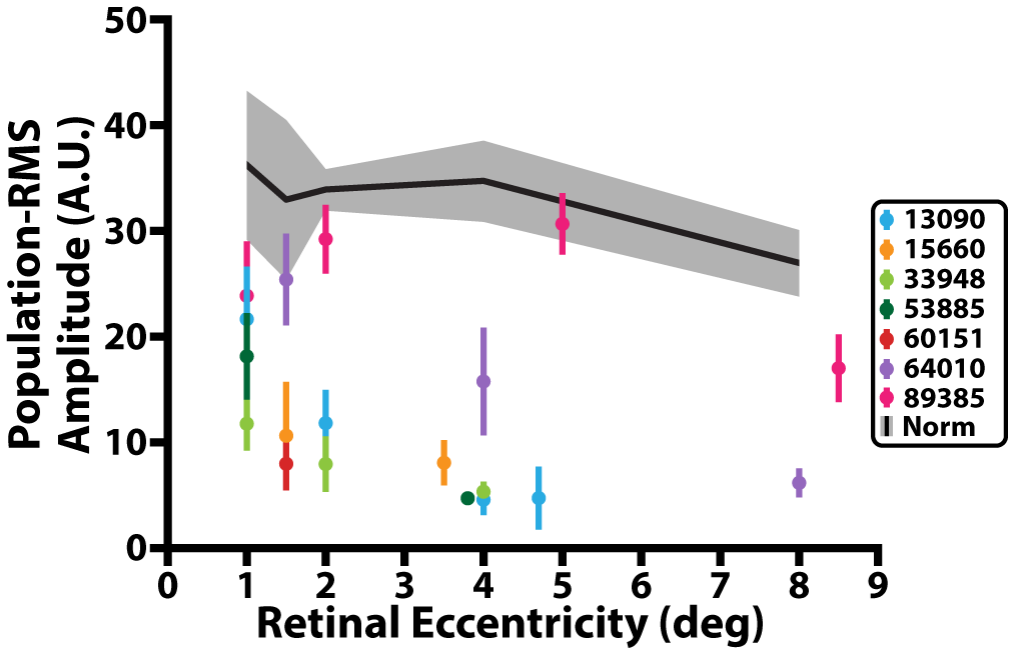
iORG population-RMS amplitude across retinal eccentricity. Black line represents average population-RMS amplitude across retinal eccentricity for all control participants. Grey shading represents 95% confidence interval of the control data. Colored dots represent mean population-RMS amplitude for a given retinal eccentricity across all stimulus trials from individuals with RP. Colored lines represent 95% confidence interval of the population-RMS amplitude for a given eccentricity across all trials for each individual with RP.

**Figure 4:**
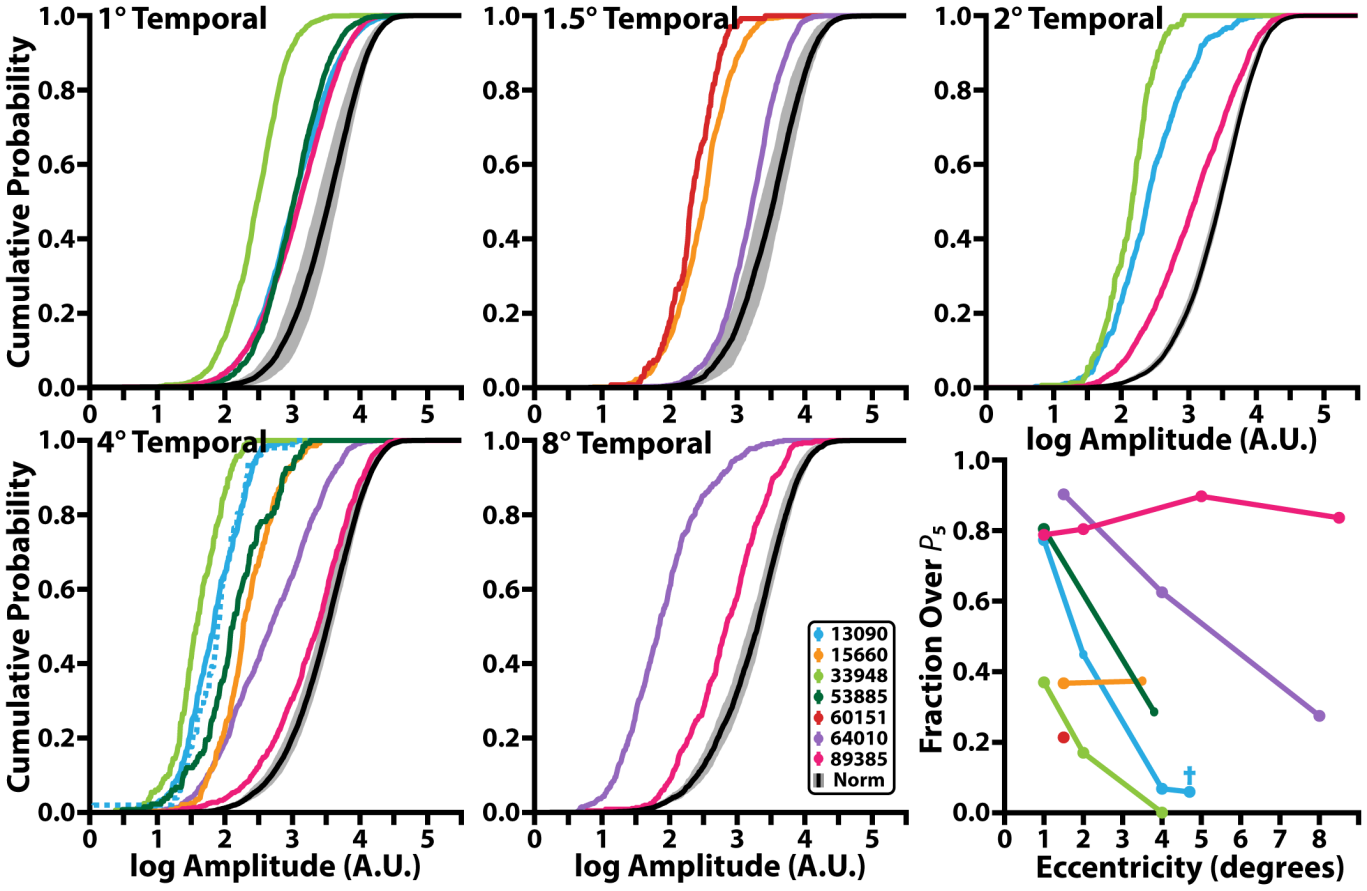
Cumulative probability function of iORG individual-RMS amplitudes across retinal eccentricity and the fraction of individual-RMS amplitudes that are greater than the 5^th^ percentile of the control individual-RMS amplitudes. Black line denotes the mean and grey shading represents the 95% confidence interval of individual-RMS iORG amplitudes for all control participants. Colored lines represent different individuals with RP. Blue dotted line in the 4° temporal plot and blue cross in bottom rightmost plot represents the edge of the lesion for 13090 which was collected at 4.7° temporal but was grouped with the 4° temporal location.

Across all 15 normative control participants, we observed ~2 times larger confidence intervals in iORG population-RMS amplitudes at retinal eccentricities closer to individuals’ PRL (1, 1.5°) than at greater eccentricities (4, 8°). We also observed a general decline in population-RMS amplitude with eccentricity, consistent with previous reports (29) using pORGs (**Figure 3**, black line). This relationship was not strictly monotonic in all of our participants, and most individuals exhibited a lower population-RMS amplitude at 1.5 or 2° relative to 1 or 4° (n=10; **Figure 3** black). This was mirrored in iORG individual-RMS amplitudes (**Figure 4**, black lines with gray shading).

Nearly all individuals with RP showed a significant reduction in population-RMS amplitudes relative to controls at similar eccentricities (**Figure 3**). Of the two individuals without consistently reduced amplitudes, one (64010) was for a location nearest the PRL only, and the other (89385) had two locations inside the normative 95% CI (4° and 8°). Across all participants with RP, population-RMS amplitudes closer to the individuals’ PRL were greater than those closer to the edge of each participant’s TZ.

Like population-RMS amplitudes, the cumulative density functions of individual-RMS amplitudes showed an overall reduction in healthy responding cones in all individuals with RP apart from 89385 (**Figure 4**). These distributions allow us to further interrogate what fraction of the cones are functioning similar to our normative population. As our stimulus wavelength does not significantly stimulate S cones, we expected S cones to represent the lowest amplitude in each population. Thus, based on the average S cone percentage (56–61), we used the 5^th^ percentile of our normative population to determine the proportion of putative healthy cells in all participants with RP. In this analysis approach, a fully ‘healthy’ cone mosaic would thus have ~95% of its cells over this threshold. The proportion of putative healthy cells is reduced in all participants with RP (mean: 63.7%; range: 14.2%-92.5%); most participants with RP have a higher proportion of healthy cone amplitudes closer to their PRL (72.8 ± 16.0%<=2°) and have a lower proportion of healthy cone amplitudes closer to their TZ (53.5 ± 28.6% >2°; **Figure 4**, bottom right). Excluding extrema from 89385 lowers the mean to 70.1 ± 16.0% for<=2°, and 42.5 ± 21.0% for all locations >2°.

### 3.4 The relationship between cone structure and population-RMS

**Figure 5:**
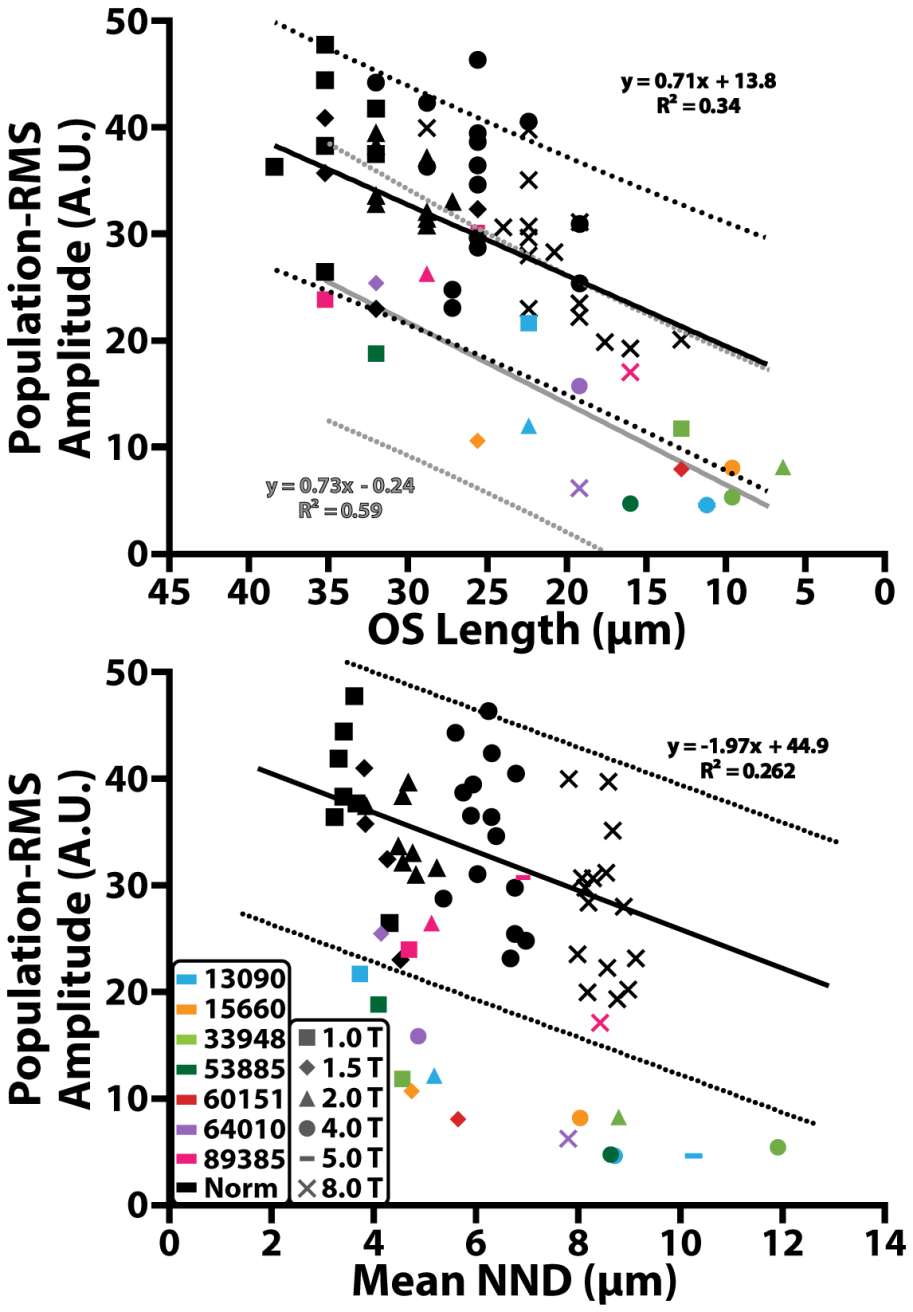
Population-RMS amplitude across cone outer segment (OS) length and mean nearest neighbor distance (NND). Black solid lines represent a linear regression of the control data. Black dotted lines represent the 95% prediction interval of the control data. Gray solid lines and dotted line correspond to the 95% prediction interval of data from individuals with RP. Colored shapes represent data from individuals with RP and black shapes represent data from the control cohort. Different retinal eccentricities are labeled using different shaped markers.

Across all control participants, population-RMS amplitude decreased monotonically with decreasing cone outer segment length (**Figure 5**, black, top panel) and increasing mean nearest neighbor distance (**Figure 5**, black, bottom panel), consistent with previous observations using phase-based ORGs (29). A significant positive linear relationship was observed between population iORG amplitude and cone outer segment length (y = 0.71x + 13.8; r^2 =^ 0.34; p<0.0001), and a significant negative linear relationship was observed between population-RMS amplitude and NND (y = -1.97x + 44.9; r^2 =^ 0.262; p = 0.0005).

In most individuals with RP there was a significant reduction in population-RMS amplitude as a function of cone outer segment length (y = 0.73x -0.24; r^2 =^ 0.59; p<0.001; **Figure 5**, gray line, top panel) and mean nearest neighbor distance (**Figure 5**, bottom panel), which fell outside the prediction intervals of the normal controls. 89385 (**Figure 5**, pink) had population-RMS values near the control group for all outer segment and mean nearest neighbor distances. One other individual with RP, 64010 (**Figure 5**, purple), had population-RMS amplitude, cone outer segment length, and mean nearest neighbor distance values that were close to that of the control group at their 1.5° temporal location. Overall, population-RMS amplitudes in RP patients were closer to controls for longer cone outer segment lengths, and lower mean nearest neighbor distances (consistent with locations closer to the fovea).

### 3.5 iORG RMS and clinical measures of retinal function

**Figure 6:**
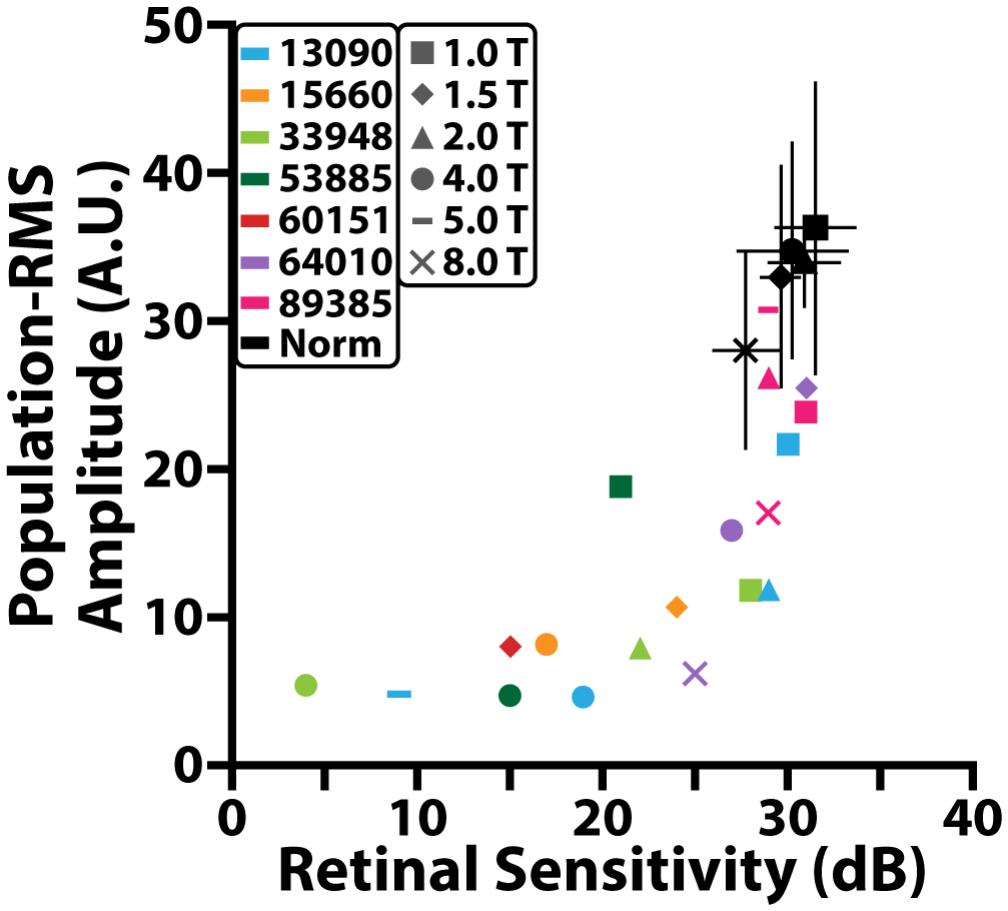
Comparison between population-RMS amplitude and MAIA retinal sensitivity (dB). Black markers represent different locations for the control cohort and black error bars denote standard deviation. Colored markers represent different locations for individuals with RP.

A significant correlation between population-RMS amplitudes and MAIA retinal sensitivity was observed across all control participants (Spearman r = 0.45, p<0.01). MAIA retinal sensitivity also decreased as a function of retinal eccentricity (**Figure 6** **, black**).

In individuals with RP, population-RMS amplitudes and MAIA retinal sensitivity were also correlated (Spearman r = 0.66, p< 0.001). Both population-RMS amplitude and MAIA retinal sensitivity were reduced on average in individuals with RP when compared to controls (**Figure 6**). Some individuals had retinal sensitivities that fell within a standard error of the controls at a given retinal location even though all but one of these locations had population-RMS amplitudes that were lower than the control cohort.

## 4 Discussion

In this study, iORGs were used to assess photoreceptor function in individuals with retinitis pigmentosa and a control population of individuals with no reported retinal pathology. We compared iORG RMS amplitudes in these individuals to measurements of cone structure such as nearest neighbor distance and cone outer segment length and compared iORG RMS to a gold standard clinical measure of retinal function, microperimetry (MAIA). Structural metrics were consistent with previously published values for controls and individuals with RP (50, 62, 63), and we observed higher NND values for individuals with RP at more eccentric locations, consistent with the progression of RP. Additionally, MAIA microperimetry indicated relatively preserved function near many of the RP individuals’ foveae. Like metrics of structure, these results were variable across individuals, consistent with previous reports of varied phenotypes and disease progression across different individuals with RP (64, 65).

For most RP participants, average population-RMS amplitudes were far lower than our control population, with two notable exceptions: 1.5° in 64010 and 2° and 5° in 89385. 64010 has a less severe RP phenotype, and cones close to her fovea have markedly preserved structure and function, though amplitude still declines with eccentricity. However, 89385’s iORG pattern is not consistent with previous reports of functional losses in RP (10, 65, 66) despite their clinical RP diagnosis; indeed, they showed decreased RMS amplitudes at 1°, a normal RMS amplitude at 5°, and another decrease near 8°. While not detected by the NND metric, the structure of 89385’s photoreceptor mosaic appears grossly abnormal (**Supplementary Figure 3**), with focal patches of missing rod photoreceptors and preserved cones. Thus, while our data is not consistent with a “classical” definition of RP, we cannot rule out other possibilities such as mosaicism (67), which has been reported in individuals with RHO mutations (68, 69). Further, without genetics we cannot rule out other diseases with pigmentary deposits, such as pigmented paravenous retinochoroidal atrophy (70), or even congenital rubella (71). Unfortunately, we were not able to collect a genetic sample from this individual to confirm either hypothesis.

We also interrogated how many cones in each area had putatively “healthy” (e.g. greater than the 5^th^ percentile of normal cones) individual-RMS amplitudes. This analysis allowed us to determine if cone function in a region is lost uniformly, or if certain cells retained normal function while others declined. Interestingly, three individuals who had substantially reduced population-RMS amplitudes at 1° (13090, 53885, 89385) had ~80% of their individual-RMS amplitudes within normative ranges, suggesting that their low population-RMS amplitudes were being driven by a small subset of cones in those ROIs. The individual-RMS amplitudes of 89385 again stand out as unique compared to other individuals with RP, at the 1° and 2° temporal location there are less cells responding normally than at the 5° and 8° temporal locations. Overall, the individual-RMS analysis highlights that near the fovea there are more cells with normal responses in most individuals with RP compared to their more temporal imaging locations. Perhaps most importantly, this indicates that even in areas with reduced function there are healthy cells remaining that could benefit from maintenance treatments to preserve/prolong their function.

One of the most interesting aspects of these results is the relationship between structure and function in these individuals. When comparing iORG population-RMS amplitude against the NND distance of the same cells, we observed a largely linear relationship in our normal controls, similar to that previously observed by Jiang et al. (29). However, this relationship changes significantly in individuals with RP, taking on the appearance of exponential decay as a function of NND. By extension, this indicates that very small changes in NND correspond to large changes in iORG amplitude in individuals with RP, plateauing at what appears to be the noise floor around ~5 AU, and it appears that the largest amount of change occurs over a NND range of only 2 microns.

The relationship between cone outer segment length as measured through Cirrus OCT and iORG population-RMS was less clear. Typically, individuals with RP had lower population-RMS amplitudes for a given cone outer segment length than controls, however there were numerous instances where the population-RMS amplitude for a given outer segment length from individuals with RP fell within the range of controls, and the prediction intervals for the two populations overlapped. There were several instances where individuals with RP had outer segment lengths that were the same as controls, but with reduced iORG amplitude and the regression for our participants with RP had a slightly steeper slope but had a substantially decreased y-intercept. This disconnect between outer segment length and amplitude in individuals with disease is consistent with prior observations using pORGs (44) and suggests that outer segment length alone can’t account for the observed reductions in amplitude. However, as our Cirrus OCT that lacks the transverse and axial resolution necessary to interrogate the OS length of individual cones, our OS length measurements were in effect an aggregate measurement and may therefore underestimate the remaining OS.

Finally, we compared iORG population-RMS amplitude to a gold standard for assessing cone function, microperimetry (MAIA). For many imaging locations, both reduced population iORG amplitude and retinal sensitivity were observed in individuals with RP when compared to controls. There was some overlap in retinal sensitivity between controls and some individuals with RP, for example the 1.5° location of 64010, the 1° temporal location 13090, and both the 1° and 2° temporal locations fell within a standard error of the retinal sensitivity of controls. Like NND, we observed an exponential relationship between retinal sensitivity and population iORGs, wherein a very small change in retinal sensitivity (<4dB) corresponded to a very large change in population iORG amplitude (>20AU). However, the dynamic range of the two assays were substantially different, with the iORG reaching its noise floor long before the MAIA (between 15–20dB).

This study had some limitations and potential biases that should be considered when interpreting its results. As mentioned above, the presence of cataracts and/or macular edema precluded successful AOSLO imaging in two individuals with RP. While we excluded individuals with cataracts to ensure our full stimulus was reaching the retina, nearly all participants had microedema and/or regions with poor image quality, and in some cases individual cones were unresolvable or ambiguous within the attempted imaging locations (especially in the peripheral retina). The split detection modality was used to aid in visualizing cone inner segments, but in cases where cones were still unresolvable, even with split-detection, that location was omitted from further analyses (53885, 60151). However, omitting individuals or locations with advanced disease can lead to selection bias at the population level, and future work using stimuli with varied imaging entrance locations (to avoid cataracts), or an ORG modality that is nominally less sensitive to inner retinal edema (e.g. AO-OCT) has the potential to reduce this source of bias. Further, as noted above, RP is a group of inherited retinal diseases that encompass many different genetic mutations (2, 72) and exhibit variable disease progression (10, 65, 73) and phenotypes (62). However, this study includes only seven individuals with RP (only 4 of which had genetic information) and is unlikely to be representative of all RP subtypes. Future genotype-specific work will be necessary to establish an iORG “signature”, if any, from the many RP subtypes.

While all efforts were made to maintain a consistent stimulus dose to the retina, inter-individual variability in ocular biometry means that the photons/µm^2^ delivered to each cone varies slightly between individuals. To assess its contribution to the iORG variability observed in this study, the stimulus photon density was calculated for all participants using the model published by Jiang et al. (29) and was plotted against the population-RMS amplitude of each participant (**Supplementary Figure 2**; data mean subtracted for visualization). The average stimulus photon density at the retina was 2.33x10^7^ photons/µm^2^ (range = 1.86x10^7^ to 2.77x10^7^ photons/µm^2^). There was no significant difference in stimulus photon density between controls and individuals with RP (Student’s t-test, p = 0.475). A linear regression model was fit to the data and no significant relationship was found between mean subtracted population-RMS amplitude and stimulus photon density at the retina (y = 10–^7^x – 2.58; r^2 =^ 0.0014; p > 0.05). Of note, this model does use a linear retinal magnification factor and doesn’t factor in individual variations in absorptance of ocular structures (cornea, lens) and may exhibit nonlinearities on the extrema when factoring in more complex optical models of the eye (74, 75).

Overall, we found the differences between the iORG and MAIA to be quite interesting and a microcosm of the differences between psychophysical assays that integrate information across the entire visual system (microperimetry), and objective, cell specific assays such as optoretinography. For instance, we observed iORG RMS rapidly approaching and reaching its noise floor around 15–20dB in retinal sensitivity, implying that cones with sensitivities beneath these values are functioning so poorly that there is no optical path length change in these cells, and thus no iORG. However, assuming the participant is attending to their fixation task during microperimetry, there remains retinal sensitivity at this location, indicating that there remains at least some residual function such that the spot can be seen by the participant. Importantly, these data underscore the limitation of what optoretinography measures in this paradigm: path length changes in the cone outer segment. Thus, while optoretinography appears to be closely related to cone function in the early to moderate stages of disease, when a cell reaches a stage where the OS is disorganized or not present, the assay may no longer provide a sensitive assessment of cone health. In many ways, this sensitivity seems a boon for the iORG- and indeed, significant deficits were detected in regions with ostensibly normal retinal sensitivities. However, it does suggest caution in interpreting results from future optoretinography studies of other retinal diseases, as the absence of an ORG signal does *not* necessarily indicate that the cell is non-functional- it only means that there isn’t a detectable change in the cone outer segment.

Overall, these data suggest that microperimetry and measures of cone structure, at present, remain the best method for detecting complete cell loss, but optoretinography appears to be most sensitive to the earliest changes in cell function. Detecting these early changes in cell function may be valuable as either a screening tool, or for assessing the efficacy of therapeutic interventions.

## Data availability statement

The raw data supporting the conclusions of this article will be made available by the authors, without undue reservation.

## Ethics statement

The studies involving humans were approved by Institutional Review Boards at the Medical College of Wisconsin (PRO00038673). The studies were conducted in accordance with the local legislation and institutional requirements. The participants provided their written informed consent to participate in this study.

## Author contributions

MG: Conceptualization, Data curation, Methodology, Software, Writing – original draft, Writing – review & editing, Formal analysis, Visualization. TC: Data curation, Resources, Supervision, Writing – review & editing, Investigation. RC: Data curation, Resources, Supervision, Writing – review & editing, Conceptualization, Formal analysis, Funding acquisition, Methodology, Software, Validation, Writing – original draft, Visualization.

## Supplementary material

Supplementary Figure 1 Confocal and split detection AOSLO images of the photoreceptor mosaic in one representative control and individual with RP. Confocal (top) and split detection (bottom) images across different retinal locations for one control (23045) and one individual with RP (13090). Images cropped to a 0.5° by 0.5° area.

Supplementary Figure 2 Mean subtracted population-RMS amplitude as a function of stimulus photon density at the retina. Blue dots denote different study participants. Dotted blue line represents the line of best fit from a linear regression.

Supplementary Figure 3 The fundus image (left), confocal (top), and split detection (bottom) AOSLO images of the photoreceptor mosaic in 89385. Images cropped to a 0.5° by 0.5° area for visualization.
