# Supplementary figures and images for: Intensity-based optoretinography reveals sub-clinical deficits in cone function in retinitis pigmentosa
Source: Front Ophthalmol (Lausanne). 2024 Jun 4;4:1373549. doi: 10.3389/fopht.2024.1373549 (PMC11182324; doi:10.3389/fopht.2024.1373549)

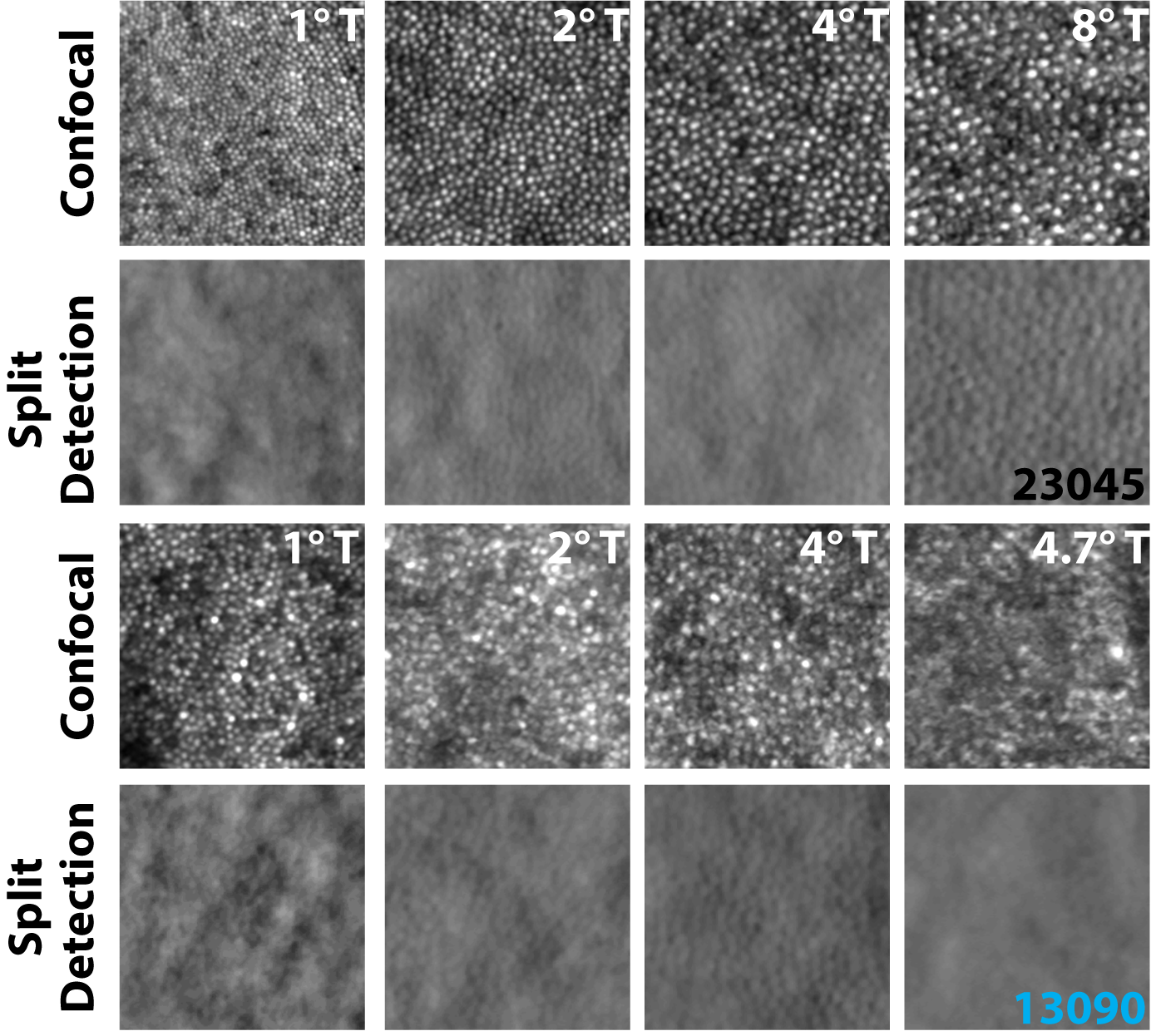

Supplement: Supplementary Figure 1 — Confocal and split detection AOSLO images of the photoreceptor mosaic in one representative control and individual with RP. Confocal (top) and split detection (bottom) images across different retinal locations for one control (23045) and one individual with RP (13090). Images cropped to a 0.5° by 0.5° area. [file Image_1.tif]

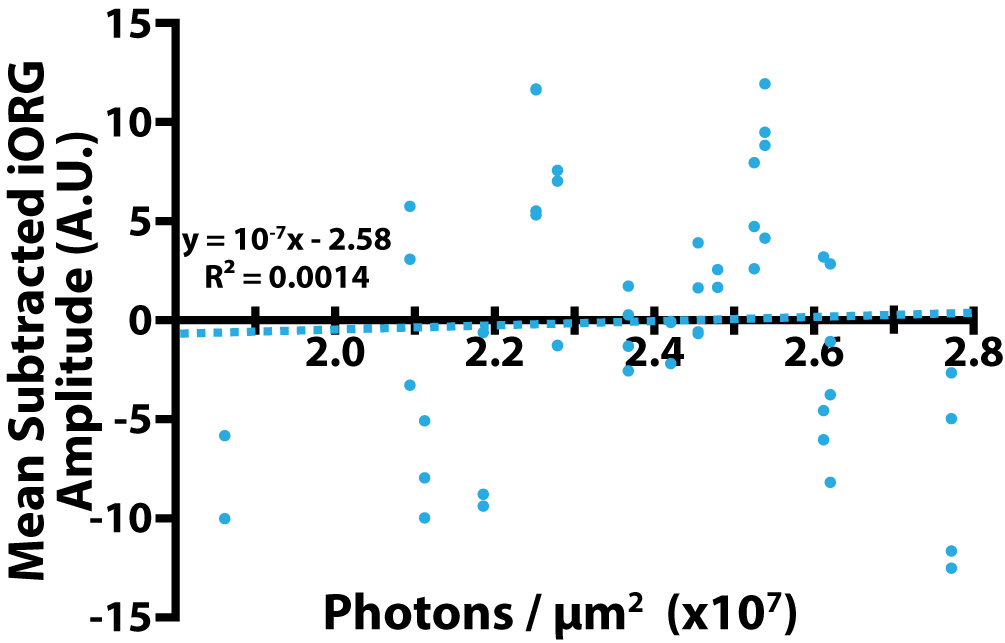

Supplement: Supplementary Figure 2 — Mean subtracted population-RMS amplitude as a function of stimulus photon density at the retina. Blue dots denote different study participants. Dotted blue line represents the line of best fit from a linear regression. [file Image_2.tif]

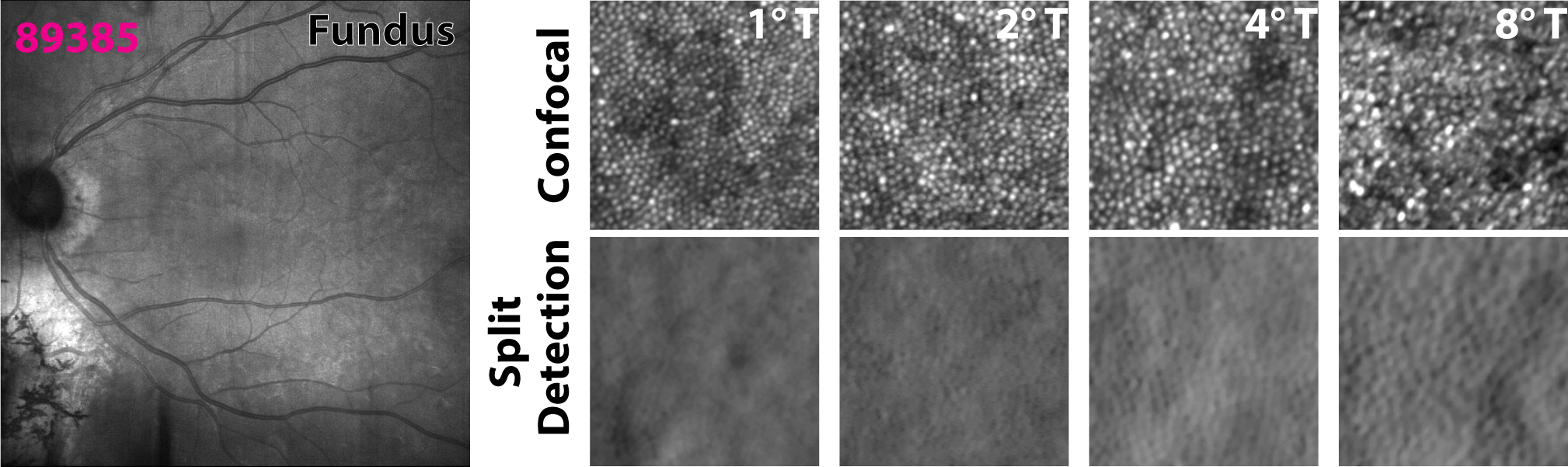

Supplement: Supplementary Figure 3 — The fundus image (left), confocal (top), and split detection (bottom) AOSLO images of the photoreceptor mosaic in 89385. Images cropped to a 0.5° by 0.5° area for visualization. [file Image_3.tif]
